# Suicidal Risks in Reports of Long-Term Treatment Trials for Major Depressive Disorder

**DOI:** 10.1093/ijnp/pyv107

**Published:** 2015-09-19

**Authors:** Ross J. Baldessarini, Wai Keat Lau, Jordan Sim, Min Yi Sum, Kang Sim

**Affiliations:** Department of Psychiatry, Harvard Medical School, Boston, MA (Dr Baldessarini); International Consortium for Psychotic and Mood Disorders Research, McLean Hospital, Belmont, MA (Drs Baldessarini and K Sim); Yong Loo Lin School of Medicine, National University of Singapore (Drs Lau, J Sim, and K Sim); Research Department (Dr K Sim and Ms Sum), and Department of General Psychiatry (Dr K Sim), Institute of Mental Health, Singapore.

In our recent review and meta-analyses of reports of long-term trials of treatments for major depressive disorder patients, we noted that remarkably little potentially relevant and interesting clinical information was included in most reports (Sim et al., 2015). This limitation seemed particularly remarkable in long-term studies carried out at great effort and expense. In general, reports of clinical trials in various disorders appear to focus rather narrowly on outcomes for which the trials were designed, usually rates of relapse or recurrence of a particular illness (Baldessarini, 2013).

In an effort to evaluate this impression more closely, we re-reviewed the reports cited in our review (Sim et al., 2015), focusing specifically on any indications of suicidal behaviors, which are of particular interest with major mood disorder patients. We recorded any comment or data pertaining to suicidal events, including completed suicides, attempts or self-injuries, or suicidal ideation typically significant enough as to require removal from the trial. Exposure times include not only the long-term phases of the trials but also lead-in weeks with placebo treatment as well as initial open-label phases of antidepressant treatment prior to randomization to continue active treatment or discontinue to placebo, with categorization based on the treatment assigned during the long-term phases of trials.

Very few reports included information concerning suicidal risks, even broadly defined. In all, we found 12 such publications among 85 (14.1%) reviewed reports. Reports with any information about suicidal events included 6 placebo-controlled continuation trials for up to 1 year, 6 antidepressant trials lasting more than 12 months, and no trial of a psychosocial treatment (Sim et al., 2015) (Table 1). Most of the 12 reports provided only brief comments as to whether or not suicides or attempts occurred in active treatment or control arms of the trials, most of which had attempted to exclude currently suicidal subjects. The very low yield of 14.1% of trials reporting any information about suicidal events during the trials was surprising.

**Table 1.** Suicidal Events in Long-Term, Controlled Treatment Trials for Major Depressive Disorder

Study	Treatment	Exposure (years)	Subjects		Suicidal Events: Ideation/Attempts/ Suicides		Incidence (events/ 100 person-years)	
			Treatment	Placebo	Treatment	Placebo	Treatment	Placebo
Doogan and Caillard 1992	Sertraline	1.17	185	110	0/3/0	0/1/0	3/216.5 (1.39)	1/128.7 (0.78)
Montgomery and Dunbar 1993	Paroxetine	1.15	68	67	0/0/1	0/0/0	1/78.2 (1.28)	0/77.0 (0.00)
Versiani et al., 1999	Reboxetine	1.08	145	141	0/0/2	0/0/0	2/156.6 (1.28)	0/152.3 (0.00)
Rouillon et al., 2000	Milnacipran	1.48	104	110	0/8/2	0/5/1	10/153.9 (6.50)	6/162.8 (3.69)
Schmidt et al., 2000	Fluoxetine	0.73	379	122	1/0/0	1/0/0	1/276.7 (0.36)	1/89.7 (1.12)
Thase et al., 2001	Mirtazapine	0.98	76	80	0/2/0	0/0/0	2/74.5 (2.68)	0/78.4 (0.00)
Kornstein et al., 2006	Escitalopram	1.46	73	66	0/0/0	0/1/0	0/106.6 (0.00)	1/96.4 (1.04)
Keller et al., 2007	Venlafaxine	2.65	43	86	0/0/0	1/0/0	0/114.0 (0.00)	1/227.9 (0.44)
Kocsis et al., 2007	Venlafaxine	1.65	164	172	1/0/0	1/0/0	1/270.6 (0.37)	1/283.8 (0.35)
Kelin et al., 2010	Duloxetine	1.62	64	60	0/0/0	0/0/0	0/103.7 (0.00)	0/97.2 (0.00)
Liebowitz et al., 2010	Quetiapine	1.48	391	385	9/0/0	1/0/0	9/578.7 (1.56)	1/569.8 (0.18)
Rosenthal et al., 2013	Desvenlafaxine	0.92	272	276	5/5/0	0/0/0	10/2720 (0.37)	0/253.9 (0.00)
Totals/Means [95%CI]	(12 trials)	1.36 [1.05–1.68]	1964	1675	16/18/5 (39)	4/7/1 (12)	39/2671 (1.46) [1.03–2.00]	12/2278 (0.53) [0.27–0.92]

The observed incidence rate ratio (IRR for suicidal events/100 person-years) indicates 2.77-fold [CI: 1.42–5.82] greater with antidepressants vs placebo (exact *P*=.0005). Observed incidence (%/y) with antidepressant treatment vs placebo (based on N and time from reporting studies only) was: ideation (1.11 vs 0.32), attempts (2.48 vs 1.79), suicides (1.28 vs 0.61), and suicidal acts (attempts+suicides: 2.03 vs 1.44; corresponding IRR [with 95%CI] were: ideation (3.45 [1.11–14.2]; *P*=.009), attempts (1.39 [0.55–3.94]; *P*=.24), suicides (2.08 [0.23–98.2], *P*=.28), and suicidal acts (1.40 [0.61–3.63], *P*=.21). References to trials cited are provided in Sim et al. (2015).

It is also noteworthy that the average rates of suicide attempts and suicides were very high despite efforts to exclude currently suicidal patients. The risk of suicide averaged 1.08%/year, and of attempts, 2.24%/year, across treatment arms of trials. In addition, there was an ominously low apparent ratio of attempts/suicides of 2.07, compared with >30 in the general population (Kessler et al., 2005; Tondo et al., 2007). The observed rates of attempts and suicides are far higher than in the general population and even higher than those reported in clinical samples of outpatients diagnosed with major depressive disorder, which average approximately 0.045%/year for suicide and 0.480%/year for attempts (Bostwick and Pankratz, 2000; Tondo et al., 2007). Such high levels of suicidal risk may reflect the difficulty of excluding potentially suicidal subjects from trials and the fact that trial entry required being acutely depressed in order to study treatment effects (Sim et al., 2015). Nevertheless, if the findings are valid and representative, they underscore the importance of considering suicidal risks in such trials.

Even more remarkable were indications that the overall risk of suicidal events was significantly greater in trial arms involving active, antidepressant treatment vs placebo controls when findings were pooled across all 12 trials and in 9/12 of the trials (Table 1). However, since none of the trials was specifically designed to address suicidal risk and the trials yielding data on suicidal risks were few, the summarized observations may merely represent chance findings, with risk of selective overreporting of high rates. Alternatively, they parallel the unresolved relationships of antidepressant exposure to suicidal risks (Baldessarini et al., 2007; Stone et al., 2009; Nischal et al., 2012), and surely they warrant further study.

The present findings strongly encourage the recommendation that important outcomes of clinical and research significance more routinely be included in reports of treatment trials for psychiatric disorders, especially trials involving long-term observations under controlled conditions, if only to limit biased, selective reporting of unrepresentative findings. Suicidal risk among mood disorder patients is only one example. The provocative and unexpected finding of very high reported, pooled rates of suicide and attempts and greater observed suicidal risks with antidepressant treatment than with placebo indicate the need to pursue these unexpected findings. In general, we support the research practice of including information about suicidal risk as well as other clinical measures of interest, routinely, in the design, conduct, and reporting of psychiatric treatment trials.

## Statement of Interest

None.

## References

[CIT0001] Baldessarini RJ (2013). Chemotherapy in psychiatry, 3rd edition. New York: Springer Press.

[CIT0002] Baldessarini RJ Tondo L Strombom IM Dominguez S Fawcett J Licinio J Oquendo MA Tollefson GD Valuck RJ Tohen M (2007). Ecological studies of antidepressant treatment and suicidal risks. Harv Rev Psychiatry 15:133–145. 17687708 10.1080/10673220701551102

[CIT0003] Bostwick JM Pankratz VS (2000). Affective disorders and suicide risk: a reexamination. Am J Psychiatry 157:1925–1932. 11097952 10.1176/appi.ajp.157.12.1925

[CIT0004] Kessler RC Berglund P Borges G Nock M Wang PS (2005). Trends in suicide ideation, plans, gestures, and attempts in the United States, 1990–1992 to 2001–2003. JAMA 293:2487–2495. 15914749 10.1001/jama.293.20.2487

[CIT0005] Nischal A Tripathi A Nischal A Trivedi JK (2012). Suicide and antidepressants: what current evidence indicates. Mens Sana Monogr 10:33–44. 22654381 10.4103/0973-1229.87287 PMC3353604

[CIT0006] Sim K Lau KL Sim J Sum MY Baldessarini RJ (2015). Prevention of relapse and recurrence in adults with major depressive disorder: systematic review and meta-analyses of controlled trials. Intl J Neuropsychopharmacol. In press. 10.1093/ijnp/pyv076 PMC4772815 26152228

[CIT0007] Stone M Laughren T Jones ML Levenson M Holland PC Hughes A Hammad TA Temple R Rochester G (2009). Risk of suicidality in clinical trials of antidepressants in adults: analysis of proprietary data submitted to US Food and Drug Administration. BMJ 339:b2880. 19671933 10.1136/bmj.b2880 PMC2725270

[CIT0008] Tondo L Lepri B Baldessarini RJ (2007). Risks of suicidal ideation, attempts and suicides among 2826 men and women with types I and II bipolar, and recurrent major depressive disorders. Acta Psychiatr Scand 116:419–428. 17997721 10.1111/j.1600-0447.2007.01066.x

